# From Clever Composites to Credible Technologies

**DOI:** 10.3390/membranes15110342

**Published:** 2025-11-17

**Authors:** Qieyuan Gao, Libing Zheng, Daliang Xu, Bart Van der Bruggen

**Affiliations:** 1 Department of Chemical Engineering, KU Leuven, Celestijnenlaan 200F, 3001 Leuven, Belgium; qieyuan.gao@gmail.com (Q.G.); bart.vanderbruggen@kuleuven.be (B.V.d.B.); 2 State Key Laboratory of Coking Coal Resources Green Exploitation, China University of Mining and Technology, Xuzhou 221116, China; 3 Laboratory of Water Pollution Control Technology, Research Center for Eco-Environmental Sciences, Chinese Academy of Sciences, Beijing 100085, China; 4 State Key Laboratory of Urban Water Resource and Environment, School of Environment, Harbin Institute of Technology, Harbin 150090, China

**Keywords:** composite membranes, interfacial stability, data transparency, MOF/COF-based membranes

## Abstract

Composite membranes are a hot topic in the field of membrane research. With the continuous progress of technology, its development has advanced from the application of simple copolymers to diversified material combinations. This Perspective examines why many composite membranes that excel in the lab struggle to deliver credible, durable performance at scale. Our aim is to connect four issues that are often treated in isolation—interfacial stability, manufacturability, data quality, and circular design—and to translate them into practical reporting and testing habits for the community. The novelty lies in treating “credibility” as the target function: we propose discipline-first guidelines that couple dynamic interfacial measurements with standardized long-run fouling and cleaning protocols, techno-economic and life-cycle reporting, and process-aware chemistry that fits existing hardware. We outline near-term applications in water treatment and resource recovery where drop-in formats and safer solvents already enable pilot-level operation. The future scope includes round-robin builds, FAIR data deposits, and durability metrics aligned with widely used standards for fouling potential and system benchmarking. Progress, we argue, will be measured less by first-day flux and more by what survives months of operation with uncertainty and costs on the page.

## 1. The Expansion Trap

**Figure 1:**
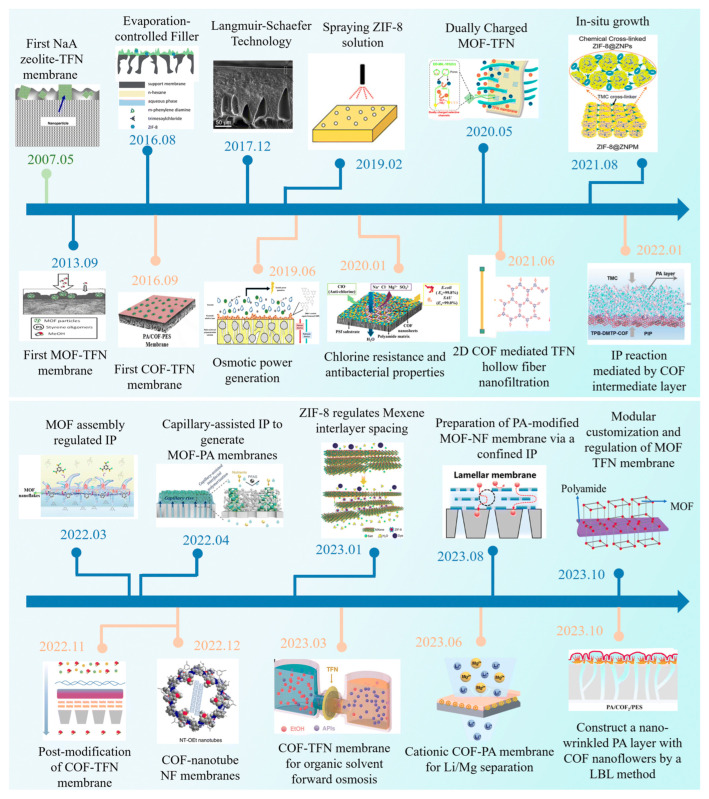
A timeline of research on MOF/COF TFN membranes prepared (Copyright 2024, Royal Society of Chemistry) [2].

Composite-membrane research has grown at a striking pace. In a few decades, it has become a busy corner of membrane science. Work that began with simple polymer blends now produces layered, multi-phase membranes. These membranes pair polymers with Metal–Organic Framework (MOFs), Covalent Organic Framework (COFs), nanocarbons, ionic liquids, and bio-based particles (Figure 1). In MOF/COF–polymer systems, the dominant early failure under water service is typically mechanical mismatch (modulus and swelling contrast that concentrates stress at the interphase), whereas chemical incompatibility (hydrolysis, oxidation, leaching) governs degradation beyond hundreds of hours, particularly under alkaline or oxidative cleaning [1,2]. Each addition promises sharper selectivity, higher permeability, or better fouling and catalytic control. Creativity has moved fast, but stability has never kept up. Many systems look strong on the bench yet fade during long runs or scale-up [2,3,4]. Across recent pilots, nominally stable thin-film composites lost 20–40% of flux or rejection within weeks once exposed to cyclic cleaning and mixed feeds; defect densities also rose during hand-to-pilot transitions as humidity and rheology control tightened. These outcomes are consistent with reports that bench recipes often miss roll-to-roll sensitivities such as evaporation rate and interfacial reaction time [5,6]. For every design that reaches a pilot line, many stay in the lab. These membranes are small, hand-cast, and tuned for one data point rather than sustained use [4,7]. Variety brings ideas, but it also brings cost. The cost penalty of “one-more-filler” designs becomes clear once TEA accounts for solvent recovery, precursor purification, and waste handling; small flux gains often vanish when normalized to USD per m^3^ treated [8]. Every new phase adds a mechanical, chemical, or thermal mismatch. Tiny voids or residual stress that seem harmless in fresh membranes can grow into cracks, swelling, or delamination under pressure cycles or chemical cleaning. Studies on MOF- and COF-based membranes point to the same lesson: service life depends less on the label and more on how interfaces are built and held together [2,4]. The barrier is not invention, but the barrier is integration [2,4,7].

Academic conventions still demand “one more”. When a design fails, the response is to add another nanoparticle, another interlayer, or another functional group. These patches can initially produce clear graphics—such as COF laminates with high screen-printing or MOF coatings with gas-selective channels—but most lose their effectiveness over time [2,3,4,9]. Durability tests expose the weak links. Interfaces that once suppressed flux become sites of swelling or plasticization. Fillers that once aided transport turn into stress concentrators or leach with pH or salinity fluctuations [2,4,7]. The same pattern is visible across all applications. In water treatment, mixed matrix and thin-film nanocomposite membranes perform well in desalination tests but fail under feeds containing surfactants, organics, or salts. After repeated cleaning cycles, flux declines and pressure drop increase due to interlayer void formation [1,3,4]. Lithium-selective membranes exhibit high Li/Mg ratios in controlled saline solutions, but few can survive months in flows rich in silica and magnesium without ligand loss or pore blockage [3,9]. In gas separation, mixed matrix membranes that appear to transcend compromise often lose their uniformity at industrial scales, while coating rheology and drying control dictate outcomes [4,7]. These setbacks do not reflect a lack of ideas. They reflect a lack of discipline. The field still prioritizes innovation over validation. A 10–20% improvement within error margins is hailed as a breakthrough. What the field now needs is slower, more stable work: prolonged anti-fouling runs, standard cleaning recoveries, adhesion fatigue tests, and leachate mass balances. These studies are unremarkable but demonstrate what endures [2,4,7]. Until these controls become routine, new claims will continue to mask fragility.

To make antifouling claims comparable, we recommend pairing any 24 h snapshot with 100–500 h continuous operation that records normalized flux, pressure-drop trajectories, cleaning recovery, and fouling potential determined by SDI or MFI-0.45 following ASTM D4189 and ASTM D8002-24. Standardization following ISO 20468-5 can link lab reports to industrial metrics. Future composite materials should be designed with simpler, more integrated layers, and employ process-aware chemistry from the outset [10,11,12]. Reducing the number of interfaces, defining the functional role of each material, and scalable process routes using certified solvents will improve reliability more than simply adding fillers. At this stage, simplicity is a sign of maturity. The question is no longer “What else can we add?” The question is “What can we remove and still meet the target?” A membrane that stays stable and reproducible for months will say more than any brief record on a permeability chart. Recent reviews of MOF and COF membranes echo this view: standardized durability metrics, controlled rheology, and transparent cost–energy reporting must accompany performance charts [3,4,7]. Only then can progress be measured not by novelty but by credibility.

## 2. Interfaces and the Illusion of Control

In composite membranes, the interface is not a line. It is a changing region where dissimilar materials must cooperate under chemical, thermal, and mechanical loads. This zone is central to performance, yet it is often the least examined. Beyond static micrographs, AFM nanomechanical mapping and micro-IR imaging can track modulus and chemistry drift across interphases during soaking or cleaning; operando TEM reveals structural change linked to local chemistry in reactive environments [13]. Apparent contact between a hydrophilic filler and a polymer can conceal mismatches in modulus, swelling, and sorption that emerge only during extended use [2]. The interface offers advantages and risks. It can speed transport, sharpen selectivity, and stiffen the film. It is also the first place to fail by delamination, cracking, or chemical attack. Most studies report only static evidence: a micrograph, a spectral shift, or a single adhesion value. Such snapshots are incomplete. Real operation drives differential swelling, interdiffusion, and solvent migration over time. Recent work shows that the interphase changes with time, and these changes—not the initial micrograph—govern service life [4]. Current test habits reinforce a false sense of control. Adhesion is often measured dry or after short soaks, with no accounting for cyclic fatigue or corrosive media. Dispersion is typically reported at low filler loadings (<5 wt%) and under ideal casting, far from industrial practice with higher solids and harsh cleaners [7]. To avoid over-estimating robustness, both dry and wet/cycled adhesion values should be reported, noting typical modulus or swelling mismatch ratios (≈10:1–30:1) between rigid fillers and hydrated polymers that drive peel-fatigue under pH or oxidant swings [1]. Interfaces that appear flawless under such conditions frequently fail once placed in service.

**Figure 2:**
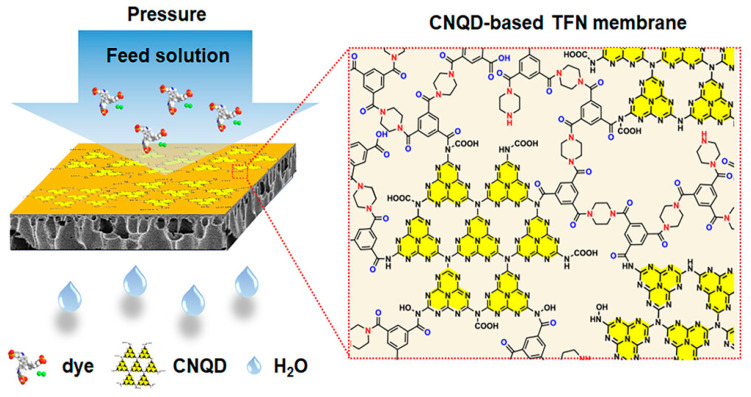
Carbon nitride quantum dot-based thin-film nanocomposite membranes (Copyright 2023, American Chemical Society) [15].

Function conflict is another pitfall. A carbon nanofiller can raise conductivity yet catalyze polymer oxidation in light or in the presence of trace metals. An ionic liquid that improves ion transport may drift under an electric field and distort the pore network. Such effects develop over hundreds of hours, beyond the window of most reports [1]. Interfaces act less like joints and more like evolving ecosystems. The task, therefore, is to engineer interphases that endure. Biology suggests routes. Cell membranes redistribute stress, repair microdamage, and reorganize domains. Analogous strategies include graded compositions, reversible bonds, and self-healing layers—approaches still uncommon but increasingly necessary (Figure 2) [9]. Finite-element and molecular-dynamics modeling now help visualize interfacial degradation and rank architectures before fabrication, while reversible/self-healing chemistries such as Diels–Alder and disulfide exchange are moving from proof-of-concept to pilot environments [14]. Methods must adapt as well. A single 24 h flux value or one fouling cycle is inadequate. Studies should track how adhesion decays, where microcracks nucleate, and how cleaning or pH cycling alters chemistry. Only with dynamic, long-term, multi-stress protocols will the interface shift from hidden liability to reliable foundation.

## 3. Scale, Cost, and the Economics of Novelty

If interfaces mark the technical limit of composite membranes, scalability has become their disciplinary one. The word facile now appears almost reflexively in papers describing new nanocomposite or thin-film syntheses. A small hand-cast membrane or spin-coated coupon is often described as “facile and scalable”, as though success on a 4 cm^2^ substrate could translate directly to industrial production. In reality, facile in the lab rarely means feasible at scale [2]. When recipes move from hand casting to roll-to-roll, yield losses and defect densities rise unless evaporation, humidity, and residence time are tightly controlled; studies of slot-die coating and ultrathin polyamide formation confirm these sensitivities [5,6,8]. Scaling up reveals dependencies that remain invisible on the bench—humidity sensitivity, viscosity drift, mixing sequence, heat-transfer gradients, or even the subtle intuition of a skilled researcher. In the lab, parameters can be nudged in real time—stop when the color turns, mix until the viscosity doubles. Continuous lines allow no such improvisation. Roll-to-roll coaters require millisecond timing and micrometre control. Small drifts in temperature or flow generate pinholes, uneven cross-linking, and delamination [7,16]. Reproducibility often collapses in the move from Petri dish to pilot line. Membranes made by interfacial polymerization or sol–gel routes look excellent in small batches but lose uniformity at scale because humidity, evaporation, and convection change the kinetics [17]. A head-to-head study of MOF–polymer membranes found order-of-magnitude differences in strength and defect density between lab and semi-industrial runs under nominally identical settings [5]. Scalability is therefore not an afterthought. It is a scientific variable that must be measured and controlled.

Economics tighten the constraint. Many “high-performance” designs depend on ultrapure fillers, high-boiling solvents, or multiple post-treatments. Costs rise while lifetime and throughput do not. When results are normalized to treated volume or energy input, the advantage often disappears. A membrane that costs ten times more must last ten times longer or run ten times more efficiently. Few do. Cost papers also omit solvent recovery, precursor purification, and waste disposal, which skews comparisons with commercial products [3,17,18]. The sticking point is mindset. Much of the literature treats membranes as prototypes, not as products. Most studies still overlook key measures of practical performance. Most studies still omit solvent-recovery efficiency. Many also skip energy use per square metre of active area. Emissions during fabrication are rarely quantified. Discussion of worker safety is even less common. Discussion of environmental compliance is rarer still—even though these factors decide whether a process can be licensed [18]. Water treatment shows the gap clearly. Casting with N-methyl-2-pyrrolidone (NMP) remains common. Fluorinated surface modifiers also remain common. Both choices now sit uneasily under tightening Registration, Evaluation, Authorisation and Restriction of Chemicals (REACH, EU regulation) rules. Replacing these chemicals after scale-up usually forces a full process redesign. Compliance pressure is steering synthesis toward safer solvent systems; for interfacial polymerization and phase inversion, cyrene and optimized aqueous/DMSO routes have delivered comparable film quality with improved EHS profiles [19]. Realism therefore belongs at the start. Many groups now treat industrial constraints as design inputs. They select chemistries that pair with approved solvents and moisture-stable precursors. They adopt slot-die, spray, and other roll-to-roll methods from day one [7,18]. Water-based dispersions look promising. Solvent-free vapor-phase polymerization looks promising as well. These routes cut hazardous waste while preserving selectivity [7]. The lesson is direct. The most valuable composites do not reinvent the factory. They run on the lines that already exist. Cost-performance comparisons (USD per m^3^ treated water) between experimental and commercial membranes clarify economic viability [8].

Scalability is more than an engineering milestone. It is a checkpoint. It requires evaluating whether new membrane technologies can be implemented within realistic material, economic, and regulatory constraints. Early focus on manufacturability avoids dead ends, as does early focus on cost. These habits break the cycle of brilliant lab data and industry silence. Lasting progress will not come from increasingly exotic chemistries. It will come from process-conscious design based on reproducibility, transparent reporting, and environmental responsibility [3,7,15].

## 4. The Data Problem and the Culture of Success

Credibility in composite membrane research comes from both academic knowledge and technical expertise. The number of papers is increasing, but reproducible, leading-edge work is rare. Many authors value neat stories more than open methods. Many readers accept clean figures in place of full datasets. Too often, correlation is treated as causation. Too often, selective plots replace uncertainty and effect sizes. At this stage, invention is not the bottleneck. Evaluation is the bottleneck. The field now needs transparent protocols, accessible raw data, and clear uncertainty budgets. The field also needs cross-lab checks and long runs that test what lasts. Without these habits, impressive materials will keep outrunning reliable proof. It is producing data that are reliable, verifiable, and reproducible by others [18]. Performance claims are routinely linked to specific design motifs: nanopores are said to accelerate transport, functional groups to improve rejection, or ionic domains to enhance conductivity. However, very few studies isolate the variables required to substantiate these claims. Improvements smaller than the experimental uncertainty are still presented as mechanistic proof. Without proper error bars, repeated measurements, or uncertainty budgets, random variation can easily masquerade as progress. Multi-lab reproducibility studies confirm that <10% “enhancements” often vanish upon replication. For water applications, performance datasets should at minimum include SDI or MFI indices and attach open metadata compliant with FAIR principles and ISO 20468-5 guidelines [10]. Several meta-analyses have now shown that reported “enhancements” below 10% in permeability or selectivity typically vanish when reproduced independently [1,15].

Characterization practices have drifted in a similar direction. Techniques once meant to explore materials—X-ray photoelectron spectroscopy (XPS), transmission electron microscopy (TEM), atomic force microscopy (AFM), attenuated total reflectance–Fourier transform infrared spectroscopy (ATR-FTIR)—are now too often drafted to confirm a preferred story. Many papers treat a minor peak shift as proof of bonding. Many also treat a change in image contrast as evidence of uniform dispersion. Basic safeguards are often missing. Authors skip calibration standards, baseline corrections, replicate spectra, and checks with orthogonal methods. One commentary noted that spectral features have drifted from measurements to badges of belief [20]. The cause is largely structural. Journals and reviewers reward visual polish more than statistical rigor. Smooth spectra and clean micrographs earn credit. Transparent error budgets do not. These incentives also suppress reports of failure. Delamination after cleaning, filler leaching, and early selectivity loss rarely appear in print. Yet such data expose fatigue, weak interfaces, and chemical instability. Other engineering fields use these “negative” datasets to build lifetime models, accelerated stress tests, and reliability curves [21]. Membrane science too often resets after each “breakthrough”. The field then repeats old mistakes under new acronyms. Inconsistent reporting deepens the problem. Many papers describe feed composition, transmembrane pressure, and shear rate loosely. Some omit these variables altogether [22]. Authors sometimes infer active-layer thickness or pore density rather than measure them. The literature becomes rich in numbers but poor in comparability. Predictive models and machine-learning tools then struggle, because they rely on clean, well-annotated inputs. There are signs of progress. The Open Membrane Data Initiative and the EU RE-MAP platform now promote shared metadata and open repositories. These tools enable side-by-side comparisons across labs. Participation remains voluntary and uneven [23]. Wider adoption will need more than infrastructure. Journals and funders must treat transparency, reproducibility, and full data disclosure as contributions. Credit should match the credit given for performance gains.

Credibility rests on treating data as evidence, not ornament. Figures must do more than show gains; they should report uncertainty, limits, variability, and where failure occurs. Methods and raw files should be accessible. With that discipline, novelty claims become secondary to results that others can reproduce—and trust. Change needs incentives, not slogans. Journals should require reproducibility statements and access to raw data with the paper. Funders should score durability, transparency, and open reporting. Programs such as the U.S. DOE Water Security Grand Challenge and the EU Horizon Clean-Water framework already favor long-term trials and third-party validation. Extending these models would align credit with accountability. Trustworthy data are the basis of lasting progress. A figure should show improvement and its uncertainty, limits, and failure modes. When records move from decoration to documentation, the field can shift from isolated claims to cumulative, reproducible science.

## 5. From Performance to Credibility

The shift now required is conceptual, not incremental. For years, the field equated progress with steeper performance curves. Higher flux. Sharper selectivity. Faster ion transport. The next phase must prize credibility over magnitude. Credibility depends on what counts as proof.

A credible composite membrane meets three linked requirements.

Operational endurance.

The membrane must function under fluctuating, fouling, and chemically harsh feeds. Idealized solutions are not sufficient. Reports should pair initial selectivity or conductivity with thermal cycling, oxidative cleaning, and mechanical fatigue. Time on stream defines utility. Cross-sector studies show that systems reporting order-of-magnitude gains at 24 h can lose ≥40% of flux or rejection within one month [2,24]. Accelerated fouling and stress-relaxation tests, as used in several European pilot programs, give a truer measure of engineering value.

Process compatibility.

The membrane must fit existing hardware. Modules, housings, and maintenance routines set real limits. Designs that need bespoke cartridges, exotic preconditioning, or restricted solvents rarely reach the market. Drop-in formats for spiral-wound, hollow-fiber, or plate-and-frame units advance faster [7]. Process-aware design, therefore, covers chemistry, form factor, pressure tolerance, and cleanability. The key question becomes: can a technician install and service the membrane safely for years?

System-level benefit.

The membrane should add value beyond separation. Systems can recover ions, harvest waste heat, or couple catalysis and sensing to reduce footprint and energy use [25]. Electro-membrane reactors illustrate this logic. They merge ion-selective layers with catalytic coatings and replace serial steps with a single unit.

Designs that meet none of these tests are ornamental, not functional.

Credibility also depends on disciplined simplicity. Added complexity multiplies failure modes. Stacks packed with fillers and interlayers often fail by debonding or by differential swelling. Lean architectures usually hold up better. Block copolymers can self-assemble into ordered domains. Graded interphases can bridge chemical contrast. Thin-film composites can rely on controlled in situ crosslinking rather than additive layering. These choices improve reproducibility and cut environmental load [25,26]. Simplicity signals maturity, not retreat. Lifecycle thinking must sit in the brief, not in the appendix. Durable membranes are designed for life after installation. They allow routine cleaning, part replacement, safe disassembly, and material recovery. A “green” claim is empty if synthesis uses high-carbon solvents or hazardous precursors. Papers should report hard numbers alongside flux and rejection. Report embodied energy, solvent-recovery rate, mass balances of valuable components, waste generated, and carbon intensity per cubic metre treated. Add echno-economic analysis (TEA), life cycle assessment (LCA), and basic environment, health, and safety (EHS) screening to the data package. Document service procedures and recyclability. New guidance, including ISO 24528:2024 for circular water-treatment technologies, offers checklists that make such reporting consistent [27].

Reproducibility must replace novelty as the unit of progress. The field advances when any capable lab can build the same membrane from standard reagents using a public, versioned protocol. Round-robin builds, preregistered procedures, and FAIR data deposits should be routine. Community projects such as the Open Membrane Benchmark already publish shared Standard Operating Procedure (SOPs) and side-by-side datasets to set common baselines [27]. Radical transparency is essential. Publish the full parts list. Release the scripts. Upload unprocessed spectra and images. Include calibration artifacts and incident reports. Work that way, and composites shift from clever demos to dependable technology. Credibility then stands on three things: staying power, fit with the process, and stewardship. Each datum is both proof and a promise. Value endurance confirmed over time, not first-week spikes. That is how busy experimentation turns into durable impact.

## 6. Concluding Perspective

Composite membranes stand at a crossroads. The field has ideas in abundance. The field lacks coherence. Many materials separate, catalyze, or sense. Far fewer do so reliably, at scale, and within rules that matter. The next gains will come from proof, not novelty. Credibility starts with restraint. Lean designs beat busy stacks. Stable interfaces beat quick wins. Process-aware chemistry beats bespoke tricks. Endurance and reproducibility must set the bar. Actionable summary: (1) Report 100–500 h runs with cleaning recovery; (2) Publish SDI/MFI with feed chemistry; (3) Include TEA/LCA metrics; (4) Release raw spectra/images; (5) Use drop-in formats and safer solvents. When labs report time-on-stream, cleaning recovery, adhesion fatigue, and leachate balance, claims begin to carry weight. When raw data and versions of protocols are public, trust follows. The work ahead is technical and cultural. Researchers should value a figure that shows lifetime, uncertainty, and failure more than a flawless snapshot. Reviewers should ask for error budgets and negative results. Journals and funders should reward shared SOPs, round-robin builds, and FAIR datasets. Prestige should come from what survives replication. Impact will depend on alignment with the industry. The design that adopts standard modules, safe solvents, and circulation processes will achieve the fastest development. Each study should combine LCA, environment, TEA, and basic EHS with flux and selectivity. A membrane that can purify water quality, recycle resources, and safely return to circulation is worth adopting.

The outlook is grim but promising. The tools are already on the table. Automated test benches enable long-term testing. Open repositories can store spectra, images, and code. Machine learning strategies can flag drift and deviations. For such analytics, models should state minimum data-quality thresholds (replicate counts, uncertainty limits, drift-detection rules) to ensure that training datasets do not mask failure precursors. Standard sustainability metrics can anchor comparisons. What’s missing is a shared discipline. If the community embraces this principle, “smart composites” will become a trustworthy technology. These materials will be durable. These materials will be suitable for real-world processes. These materials will demonstrate their footprint. Then, progress will be measured not by the rate of new announcements, but by the longevity of membranes in sustained use.

This Perspective does not present new experiments; the proposals should be validated through round-robin builds, side-by-side pilot trials, and open TEA/LCA benchmarks. Future work should quantify durability under standardized ASTM/ISO protocols and develop shared repositories for dynamic interphase data.

## Data Availability

The original contributions presented in this study are included in the article. Further inquiries can be directed to the corresponding authors.
